# Ecto-5′-nucleotidase (CD73) is a biomarker for clear cell renal carcinoma stem-like cells

**DOI:** 10.18632/oncotarget.16667

**Published:** 2017-03-29

**Authors:** Lei Song, Wenling Ye, Yong Cui, Jianzhong Lu, Yanan Zhang, Nan Ding, Wentao Hu, Hailong Pei, Zhongjin Yue, Guangming Zhou

**Affiliations:** ^1^ Medical College, Northwest Minzu University, Lanzhou 730030, China; ^2^ Medical College, Henan University, Kaifeng 475001, China; ^3^ Department of Urology Surgery, Shuyang Hospital of Traditional Chinese Medicine, Suqian 223600, China; ^4^ Institute of Urology, Department of Urology, Gansu Nephro-Urological Clinical Center, Key Laboratory of Urological Diseases in Gansu Province, Lanzhou University Second Hospital, Lanzhou 730030, China; ^5^ Department of Space Radiobiology, Key Laboratory of Heavy Ion Radiation Biology and Medicine, Institute of Modem Physics, Chinese Academy of Sciences, Lanzhou 730000, China; ^6^ School of Radiation Medicine and Protection, Medical College of Soochow University, Collaborative Innovation Center of Radiological Medicine of Jiangsu Higher Education Institutions, Suzhou 215123, China

**Keywords:** renal cell carcinoma, cancer stem cell, SILAC, cell surface biomarker, CD73

## Abstract

Identification of a specific biomarker for cancer stem cells (CSCs) is of potential applications in the development of effective therapeutic strategies for renal cell carcinoma (RCC). In this study, both the RCC cell line 786-O and surgically removed clear cell RCC (ccRCC) tissues were implemented to grew as spheroids in serum-free medium supplemented with mitogens. This subpopulation possessed key characteristics defining CSCs. We also identified that surgically removed ccRCC tissues were heterogenic and there was a subpopulation of cells that was highly stained with rhodamine-123. Based on membrane-proteomic analyses, CD73 was identified as a candidate biomarker. We further found that CD73^high^ cells were highly tumorigenic. As few as 100 CD73^high^ cells were capable of forming xenograft tumors in non obese diabetic/severe combined immunodeficiency disease mice, whereas 1 × 10^5^ CD73^low^ cells did not initiate tumor formation. During successive culture, the CD73^high^ population regenerated both CD73^high^ and CD73^low^ cells, whereas the CD73^low^ population remained low expression level of CD73. Furthermore, the CD73^high^ cells were more resistant to radiation and DNA-damaging agents than the CD73^low^ cells, and expressed a panel of ‘stemness’ genes at a higher level than the CD73^low^ cells. These findings suggest that a high level of CD73 expression is a *bona fide* biomarker of ccRCC stem-like cells. Future research will aim at the elucidation of the underlying mechanisms of CD73 in RCC development and the distinct aspects of ccRCC stem-like cells from other tumor types.

## INTRODUCTION

Renal cell carcinoma (RCC) is a form of urologic tumor with a high metastatic index and a high rate of relapse [1]. In the Heidelberg classification, clear cell RCC (ccRCC) is the most common histological subtype of RCC [2]. RCC is characterized by a high index of advanced stages at diagnosis (17% for regional/locally advanced and 16% for metastatic stage) and a high mortality (5-year survival rate for metastatic disease of only 12%) [3], and metastatic RCC is notoriously resistant to chemoradiotherapy [4]. Studies from leukemia and a number of solid tumors support the notion that cancers are maintained by a subpopulation of self-renewing and tumor-initiating cells, which are generally known as cancer stem cells (CSCs) [5–9]. Identification of ccRCC CSCs and clarification of their specific biomarkers are therefore expected to provide novel opportunities to develop new intervention strategies [10].

While the presence and distribution of CSCs in the hematopoietic system, breast, and brain have been demonstrated [11], they remain largely elusive in neoplasia of the kidneys. Previously, Lichner *et al*. [12] demonstrated the existence of CSCs in two human RCC cell lines by employing the spheroid culture system. Ueda *et al*. [13] used two different approaches to identify CSCs in RCC cell lines, the traditional Hoechst 33342 dye efflux assay and the enzymatic approach based on the evaluation of aldehyde dehydrogenase 1 (ALDH1) activity. Yamashita *et al*. [14] suggested that DNAJB8 might play a role in CSC initiation and resistance to chemotherapy of RCC. Nevertheless, to date identification of CSC still holds mystery owing to a lack of specific stem cell biomarker for RCC. It is likely that any biomarker of RCC CSCs would be distinct from those of other types of tumor. For instance, clinical significance of CD133 expression in human RCC is inconsistent and varies greatly between studies [15–20]. Higher numbers of CD133^+^ cells in RCC tissue was associated with a favorable prognosis in some studies [15, 16] but not others [17]. Bossolati *et al*. [21] proposed that the CD105^+^ stem cell population in RCC may be derived from transformed CD105^+^ bone marrow-derived MSCs or mesenchymal/stromal cells [22], rather than from the kidney itself. Moreover, this marker was found not suitable for use in all RCC cells to identify CSC [23–25]. Therefore, it is of major interest to develop new approaches in order to identify new putative renal CSCs biomarker.

Our group has recently identified, with rhodamine-123 (Rho) staining and cell sorting from human RCC cell line 786-O, that a small subset of Rho^high^ cells have stem-like characteristics [26]. Also, we found that the level of Ecto-5′-nucleotidase (CD73) expression in clinical specimens was associated with the aggressiveness of ccRCC [27]. So, in this study, we verified that CD73 expression was highly associated with positive Rho staining, and it may be a specific biomarker of ccRCC CSCs.

## RESULTS

### Spheroids formed in serum-free medium contain a CSC-like population

**Figure 1:**
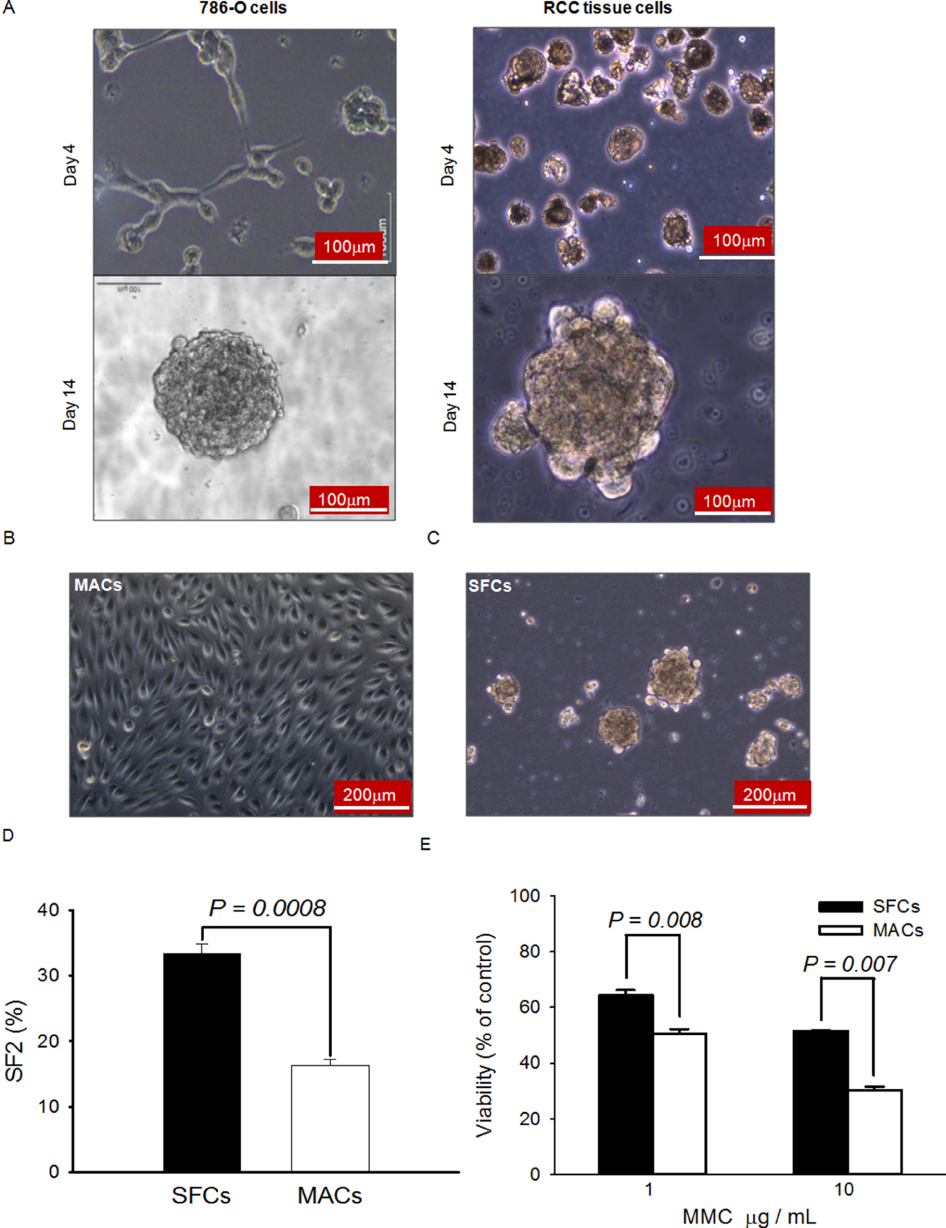
Spheroids formed in serum-free medium possess typical CSC properties (**A**) Representative morphology of 786-O cells and primary cells isolated from clinical tissue samples after 4 and 14 days of growth in serum-free medium containing mitogens. (**B**) Typical morphology of the dispersed spheroid cells when cultured in regular medium. (**C**) Typical morphology of the dispersed spheroid cells when cultured in serum-free medium. (**D**) Radiation sensitivity of spheroid and monolayer cells. A routine colony-forming assay was used to measure the plating efficiency of 100 healthy spheroid (SFCs) and monolayer (MACs) cells exposed to 0 Gy or 2 Gy of X-rays. SF2 indicates the survival fraction following exposure to 2 Gy X-rays. (**E**) Sensitivities of SFCs (filled bars) and MACs (open bars) to mitomycin C (MMC). Twenty-four hours after plating, MMC were added to reach the final concentrations indicated. After continuous exposure to MMC in culture for 2 days, the relative numbers of viable cells were assessed by MTT.

It is well known that the ability to form spheroids in serum-free medium is one of the characteristics of CSCs. To investigate whether there are CSCs in human ccRCC, 786-O cells and primary-cultured RCC cells isolated from surgically-removed tumor tissues were cultured in regular medium containing 10% FBS, or in serum-free medium containing mitogens. While the cells in regular serum grew as monolayer adherent cells (MACs), the cells in serum-free medium formed spheroids and the diameter of these spheroids reached about 200 μm in 14 days (Figure 1A). The cell cycle distributions of MACs and spheroid cells (SFCs) differed significantly. G0/G1 cells constituted 45.8 ± 5.5% of the MACs, but 71.7 ± 2.9% of the SFCs (*P* < 0.01) (Supplementary Figure 1). Dissociated SFCs could grow as MACs in medium containing 10% FBS (Figure 1B) and retained their capacity to form spheroids in serum-free medium containing mitogens (Figure 1C). The spheroids formed within 7 days in serum-free medium and have been continuously sub-cultured as spheroids for 60 passages so far, demonstrating the self-renewal and proliferative capacity of the SFCs.

To address whether the SFCs had greater tumorigenicity than the MACs, we re-suspended and inoculated cells into NOD/SCID mice. As shown in Table 1A and Supplementary Figure 2A, subcutaneous injection of as few as 500 dispersed spheroid cells successfully produced xenograft tumors in 120 days, while the same number of monolayer cells failed to generate any tumors. A larger number of MACs (5 × 10^3^ or more cells) than SFCs were required to form xenograft tumors (Table 1A). Thus, the SFCs possess greater tumor-forming capacity than their adherent monolayer counterparts. Furthermore, when mice were sacrificed 120 days after cell inoculation, we successfully isolated and cultured ccRCC cells from xenograft tumors. These tumor cells were also able to form spheroids in serum-free medium (Supplementary Figure 2B). These results suggest that a self-renewing CSC-like population persists in the xenograft tumors grown *in vivo*.

Table 1Evaluation of tumorigenicity in vivo*

**Table:** (A) Comparison of SFCs to MACs

Cell	No. of cells injected	No. of tumors/No. of mice	Mean latency, days (95% CI)	Maximum tumor volume, mm3	Day of tumor harvest
MACs	10^6^	5/5	33.8 ± 3.7	1056	90
	1.5 × 10^4^	3/4	81.1 ± 4.3	78	120
	5 × 10^3^	3/4	90.5 ± 2.1	65	120
	5 × 10^2^	0/4	‡	0	120
SFCs	1.5 × 10^4^	4/4	36.5 ± 2.1	1327	120
	5 × 10^3^	4/4	46.3 ± 4.4	1238	120
	5 × 10^2^	4/4	62.2 ± 3.8	302	120

**Table:** (B) Comparison of sorted cells based on Rho-123 intensity

Cell	No. of cells injected	No. of tumors/No. of mice	Mean latency, days (95% CI)	Maximum tumor volume, mm3	Day of tumor harvest
Rho^low^	10^7^	1/5	90.0	65	90
	10^6^	0/5	‡	0	90
	10^5^	0/5	‡	0	90
Rho^high^	10^7^	5/5	10.6 ± 2.7	1131	90
	10^6^	5/5	22.7 ± 2.6	1238	90
	10^5^	5/5	53.7 ± 4.9	1150	90

**Table:** (C) Comparison of sorted cells based on CD73 expression level

Cell	No. of cells injected	No. of tumors/No. of mice	Mean latency, days (95% CI)	Maximum tumor volume, mm^3^	Day of tumor harvest
CD73^high^	10^5^	5/5	48.2 ± 2.2	1539	120
	10^4^	5/5	55.2 ± 2.9	1056	120
	5 × 10^3^	5/5	67.2 ± 4.1	823	120
	5 × 10^2^	5/5	72.4 ± 4.6	335	120
	10^2^	2/5	89.0 ± 2.8	132	120
CD73^low^	10^5^	0/5	‡	0	120
	10^4^	0/5	‡	0	120
	5 × 10^3^	0/5	‡	0	120
	5 × 10^2^	0/5	‡	0	120
	10^2^	0/5	‡	0	120

**Table:** (D) Comparison of cells with or without CD73 knockdown

Cell	No. of cells injected	No. of tumors/No. of mice	Mean latency, days (95% CI)	Maximum tumor volume, mm3	Day of tumor harvest
Control shRNA	5 × 10^4^	4/8	48.6 ± 5.1	1175	110
	5 × 10^3^	3/8	63.0 ± 5.2	782	110
	5 × 10^2^	1/8	60	8	110
CD73 shRNA	5 × 10^4^	3/11	53.3 ± 5.8	742	110
	5 × 10^3^	1/11	62	329	110
	5 × 10^2^	0/11	‡	-	110

*Nonobese diabetic/severe combined immunodeficient (NOD-SCID) mice were used for A-C and nude mice for D. Tumor cells, as indicated, were injected in a 200 μL mixture with matrigel bilaterally into mouse inguen (one tumor per site). Sorted cells were injected within 1–2 hours after sorting. The mean latency was calculated as the time from tumor cell injection to appearance of measurable tumors (at least 5 mm × 5 mm). The 95% confidence interval (CI) was calculated for each tumor group with more than one tumor. Day of tumor harvest was determined from the day of cell injection to the determined day or when mice appeared with the syndrome of impending death. ‡ No palpable tumors were present at the day of tumor harvest.

It is generally believed that CSCs are generally more resistant to ionizing radiation and other DNA damage agents [28–31]. To verify whether RCC SFCs are more resistant to DNA damage agents, the colony-forming assay was used to compare the sensitivity of SFCs to 2 Gy of X-rays with that of MACs. As shown in Figure 1D, the survival fraction (SF2) of SFCs was 33.30 ± 1.45% and that of MACs was 16.33 ± 0.88%. The difference was significant (*P* < 0.001). The MTT assay was used to evaluate the growth inhibition of the cells treated with mitomycin C (MMC). As shown in Figure 1E, the SFCs had higher viability 48 h after exposure to MMC than the MACs. These results suggest that the ccRCC SFCs are more resistant to DNA damage agents, consistent with the notion that a CSC-like cell population exists within the spheroids.

### A subpopulation of highly rhodamine-123-reactive cells exists in ccRCC clinical specimen

**Figure 2:**
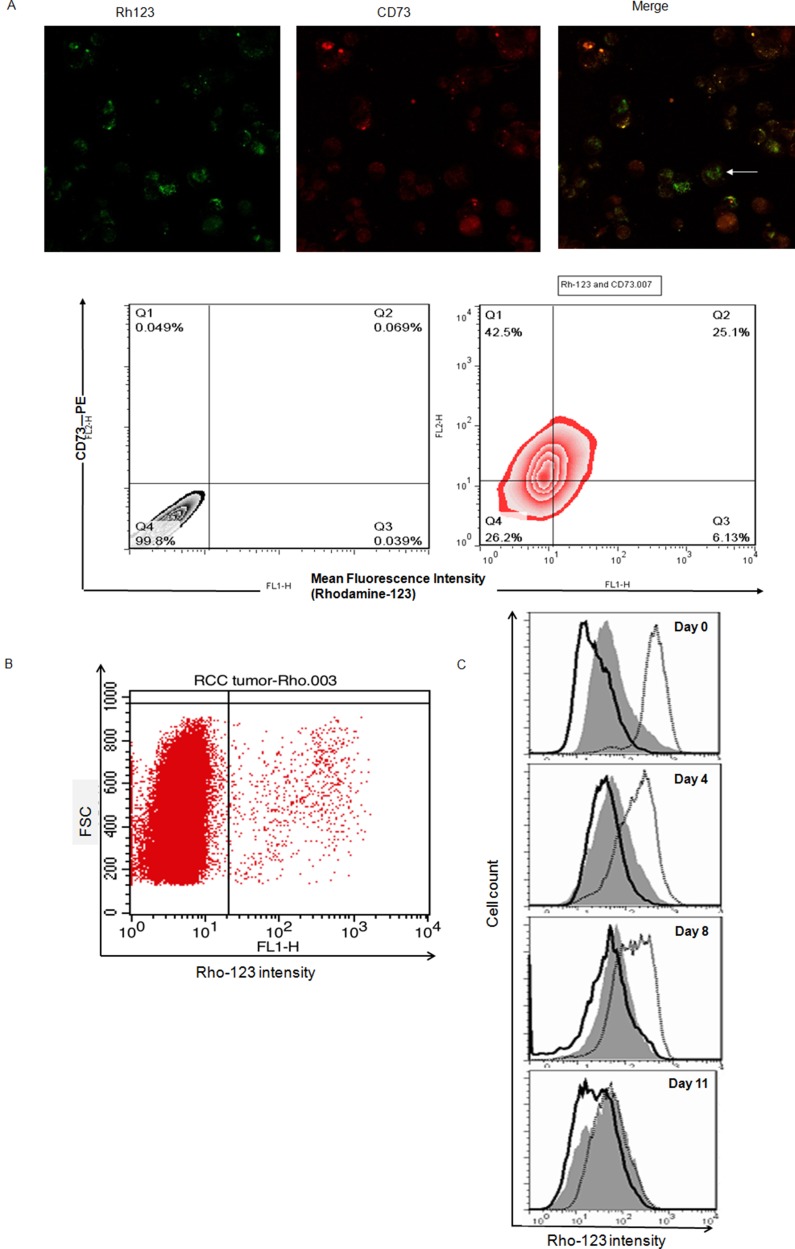
Rho^high^ cells possess CSC properties and may co-displayed with Cell Marker CD73 in ccRCC specimens (**A**) Cells co-display of Rho and CD73 (white arrow) in ccRCC, cells were labeled with Rho (green) plus PE conjugated anti-CD73 (red). Separate, as well as merged images are shown, images were taken at objective magnification ×40 and were visualized using a Leica DMI6000B microscope. ccRCC cell were analyzed for Rho staining and CD73 expression via FACS using Rho123 and anti-CD73-PE. Approximately 21.5 ± 5.9% (*n* = 6) of cells double positive for Rho and CD73-PE in specimens of ccRCC. (**B**) Rho-123 staining flow cytometry profiles of ccRCC clinical tissue samples. FSC, forward scatter. (**C**) Rho-123 fluorescence intensities after sub-culture for different times in regular medium for unsorted cells (gray), sorted Rho^low^ cells (solid line), and sorted Rho^high^ cells (dotted line). The fluorescence intensity of Rho^high^ cells was reduced remarkably during the culture period. Unsorted cells and Rho^low^ cells remained unchanged.

We used cell suspensions arise from clinical specimens to detect the co-staining of Rho and antibody CD73 conjugated PE in ccRCC (Figure 2A). The combination of the Rho123 staining approach with the CD73 staining revealed a considerable overlap between the Rho^high^ and CD73^high^ cells. A proportion of 21.5 ± 5.9% (*n* = 6) double positive for Rho123 and CD73-PE existed in specimens of ccRCC. Due to the heterogeneity, the ccRCC cells can be divided into two subpopulations, Rho^high^ and Rho^low^[26], according to Rho-123 fluorescence intensity on the flow cytometry profile for cells directly dissociated from primary ccRCC specimens (Figure 2B). The Rho^high^ subpopulation represented of the 18.8 ± 7.2% of primary ccRCC tissue cells.

To determine whether the Rho^high^ and Rho^low^ specimen cells possess differences in tumorigenic capacity, subcutaneous inoculation with 10^5^, 10^6^, and 10^7^ of Rho^high^ cells initiated tumors in all of the 15 mice tested, but Rho^low^ cell injection failed to form tumors in all but one of the 5 mice injected with 10^7^ cells (Supplementary Figure 3A, Table 1B). Rho^high^ and Rho^low^ populations were tested for asymmetric division. When the Rho^high^ cells were cultured in regular medium containing serum as a monolayer for 11 days, re-analysis of Rho^high^ cells with FACS during this period revealed that the Rho^high^ cells gave rise of both Rho^high^ cells and Rho^low^ cells, whereas the Rho^low^ cells showed no ability to transform into Rho^high^ cells (Figure 2C). These data suggest that the Rho^high^ subpopulation of ccRCC with ‘stemness’ phenotype also existed in ccRCC clinical specimens.

### Identification of the candidate surface biomarkers for ccRCC CSC by SILAC

**Figure 3:**
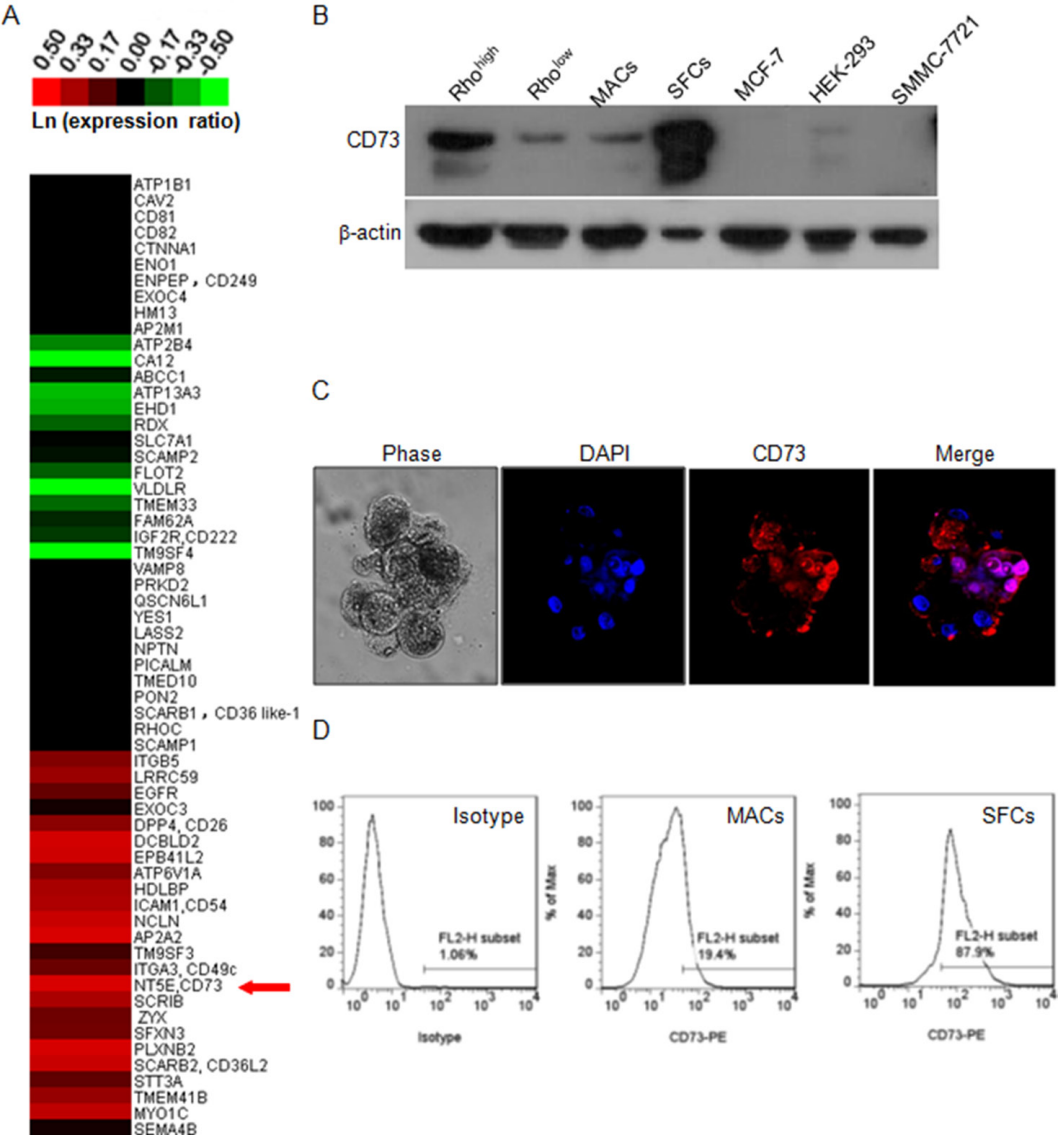
Identification of CD73 as a candidate biomarker of ccRCC CSC (**A**) Expression heat-map of a panel of 60 defined membrane proteins based on SILAC measurements. The heat-map was generated based on the relative protein levels between Rho^high^ and Rho^low^ cells and is expressed in the form of ln(ratio of Rho^high^ to Rho^low^). Magenta represents increased (compared to Rho^low^), and green represents decreased proteins. The red arrow indicates CD73 which was identified as a candidate cell surface marker of Rho^high^ cells. (**B**) Western blot of CD73 in different subpopulations of 786-O cells and three other cell lines. β-actin was used as a loading control. (**C**) Phase-contrast and fluorescent micrographs (×200, Olympus SV1000) of 786-O tumor spheroids, illustrating the high proportion of CD73-positive cells in the spheroids. DAPI was used as a nuclear counterstain. (**D**) Flow cytometry profiles of CD73 staining intensity in the mixed population (isotype) and in MACs and SFCs.

In order to identify the specific cell surface biomarkers of ccRCC CSCs, we focused on the membrane proteins differentially expressed between Rho^high^ and Rho^low^ cells by using SILAC in conjunction with LC-MS/MS. Among the 60 quantified proteins, 22 proteins were expressed at markedly higher levels in Rho^high^ cells compared to Rho^low^ cells (Figure 3A). We noticed that CD73, which is coded by *NT5E*, was among the top three highly-expressed proteins in Rho^high^ cells. Western blotting confirmed a significantly elevated CD73 expression in SFCs compared to MACs, and in Rho^high^ cells compared to Rho^low^ cells (Figure 3B). Immunofluorescent staining also revealed a concentrated expression of CD73 on the membrane of spheroid cells (Figure 3C), and a more intense staining of CD73 in SFCs compared to MACs (Figure 3D). The SFCs contained 86.1 ± 2.5% of CD73^high^ cells, while the MACs contained only 21.3 ± 2.7% of CD73^high^ cells (*P* < 0.001). These observations suggest that CD73 is a candidate biomarker of ccRCC CSCs.

### Validation of CD73 as a cell surface biomarker of ccRCC CSCs

**Figure 4:**
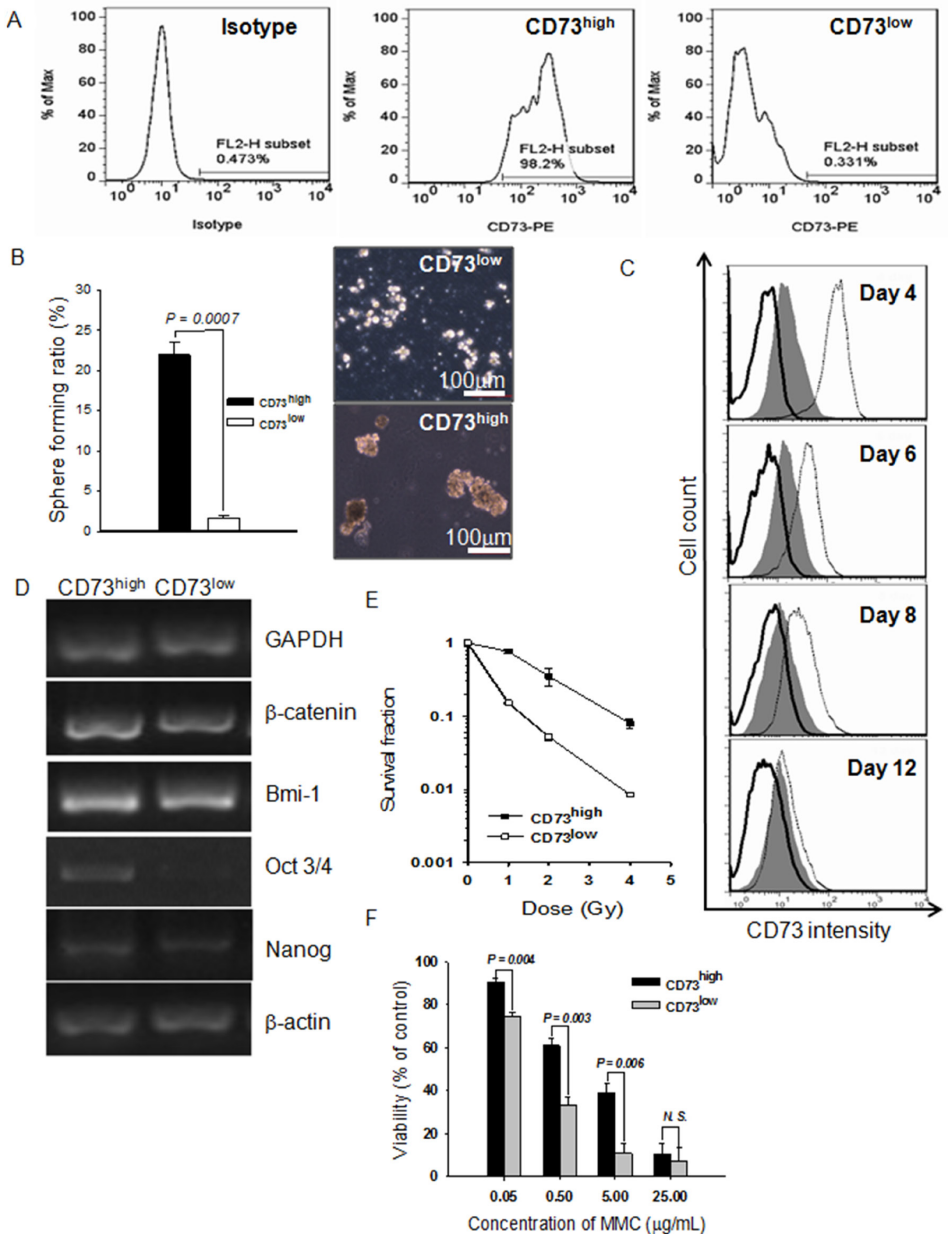
Purified cells expressing a high level of CD73 (CD73^high^) have CSC properties (**A**) Flow cytometric profile of a sorted cell subpopulation based on CD73 levels. The sorted cell subpopulations were used for profiling immediately after sorting. An isotype control was used to set the gate to define CD73^high^. (**B**) Spheroids formed by CD73^high^ and CD73^low^ cells in serum-free medium as a percentage of total cells inoculated. (**C**) CD73 expression profiles at various days after the CD73^high^ and CD73^low^ cells were cultured in regular medium. Gray fill: unsorted cells; solid lines: CD73^low^ cells; dotted lines: CD73^high^ cells. (**D**) Expression of β-catenin, Bmi-1, Oct3/4, and Nanog detected by RT-PCR. GAPDH and β-actin were used as loading controls. (**E**) Radiosensitivities of CD73^high^ and CD73^low^ cells. Sorted cells were exposed to various doses of X-rays and then the routine colony-forming assay was carried out to calculate the survival fraction. Data are shown as mean ± SEM of three independent experiments. (**F**) Growth inhibition of CD73^high^ and CD73^low^ cells by MMC, assessed by MTT assay. Results are shown as mean ± SEM of three independent experiments.

To test whether CD73 expression can be used as a valid cell surface biomarker, we sorted the cell subpopulation with high CD73 expression (CD73^high^) from 786-O cells. The purity of sorted CD73^high^ cells was more than 98% and that of CD73^low^ cells was more than 99% (Figure 4A). The same number of CD73^high^ and CD73^low^ cells were injected into the right and left inguens of NOD/SCID mice, respectively. As shown in Table 1C and Supplementary Figure 3B, injection of as few as 500 CD73^high^ cells was able to form xenograft tumors within 48 days in all the injected mice, and injection of only 100 CD73^high^ cells produced tumors in 120 days. In contrast, injection of 10^5^ CD73^low^ cells did not result in the development of any detectable tumors within 4 months.

As shown in Figure 4B, CD73^high^ cells formed new spheroids much more efficiently than CD73^low^ cells when re-cultured in serum-free medium containing mitogens. To determine the quantity of asymmetric cell division, CD73 expression levels of the initially sorted pure fractions were examined with FACS analysis. As expected, the CD73^high^ fraction regenerated CD73^low^ cells when cultured in regular medium as a monolayer, and the population became as heterogeneous as unsorted cells by 12 days, the CD73^low^ fraction showed no evidence of asymmetric division, as no occurrence of CD73^high^ cells could be identified in the population cultured with normal medium containing serum for 12 days (Figure 4C). The CD73^high^ cells exhibited higher expression of Oct3/4, β-catenin, and Nanog than the CD73^low^ cells (Figure 4D). The CD73^high^ subpopulation also exhibited significant resistance to X-ray irradiation (Figure 4E) and MMC (Figure 4F) in comparison to CD73^low^ cells. Taken together, these results show that the CD73^high^ cells possess the characteristics of CSCs, and CD73 can be considered as a specific cell surface biomarker of ccRCC CSCs.

### High CD73 expression is associated with the malignant features of ccRCC

**Figure 5:**
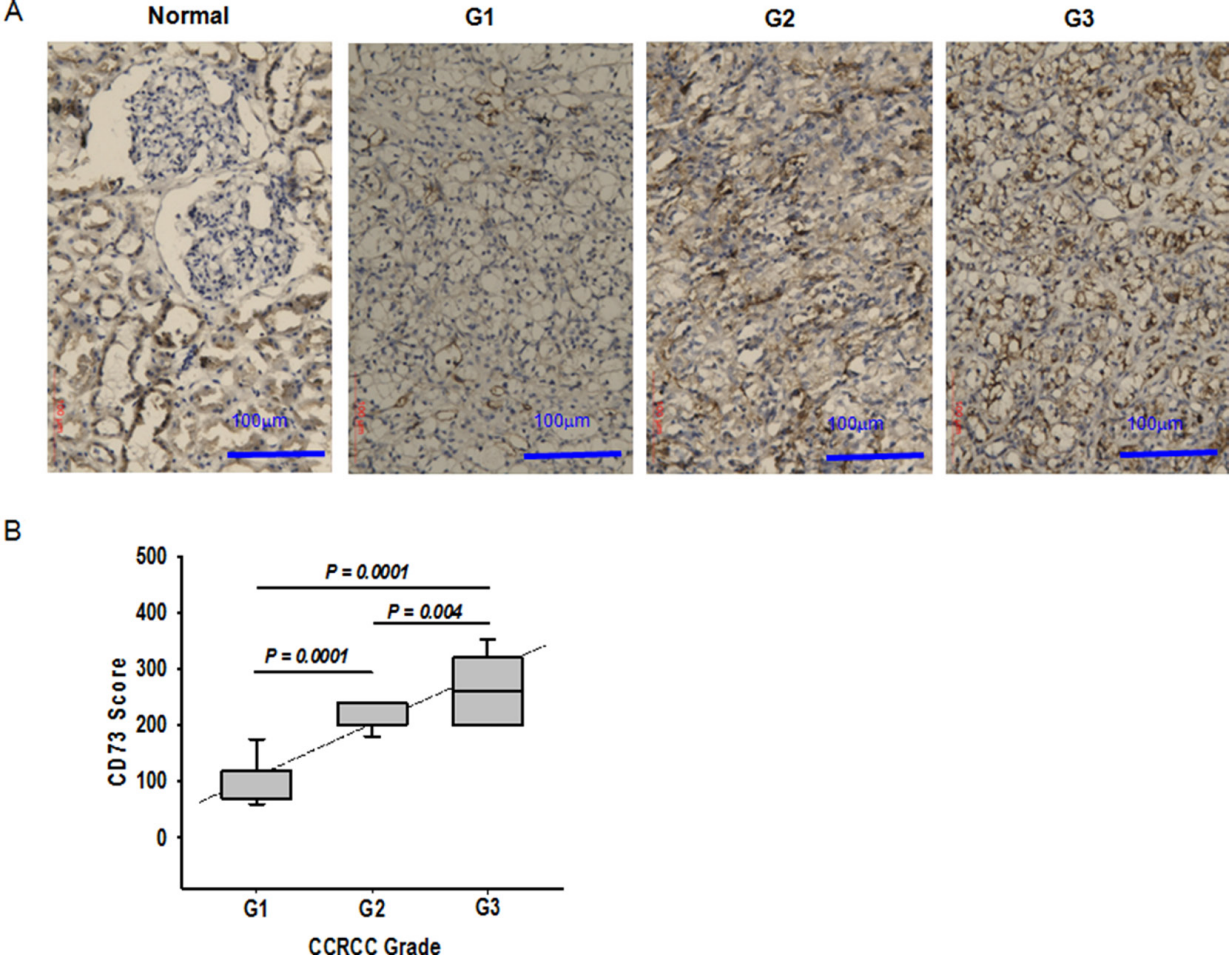
Association of CD73 expression with ccRCC progression (**A**) Immunohistochemistry of CD73 in normal kidney tissue and ccRCC tissues (Leica DMIL). G1, grade 1; G2, grade 2, and G3, grade 3. (**B**) The average level of CD73 expression in ccRCC with different grades of malignancy. Expression was assessed in 12 tissues for grade 1, 18 for grade 2, and 14 for grade 3. The CD73 score was calculated as described in Materials and Methods.

We further examined whether CD73 expression was associated with ccRCC aggressiveness. As shown in Figure 5A, little expression of CD73 was detected in kidney glomerulus whereas high expression was observed in epithelial cells of stroma and proximal convoluted tubule of normal tissues. In ccRCC specimens, the expression of CD73 was abundant in poorly differentiated vasculature. CD73 intensity in the tumor stroma positively correlates with the grade of ccRCC malignancy (Figure 5A). Among 44 cases of ccRCC examined, the expression levels of CD73 was proportional to the tumor grade of ccRCC and CD73 was more intensively expressed in high-grade G3 and G2 tumors than in low grade G1 tumors (Figure 5B). In order to further confirm that elevated expression of CD73 is associated with ccRCC aggressiveness, we repressed the expression of CD73 in 786-O cells with shRNA and then planted the cells in NOD/SCID mice to observe the tumor-forming ability. As shown in Supplementary Figure 3C and Table 1D, when the same amount of cells with control shRNA or CD73 shRNA planted to the right or left inguen of the same mouse, respectively, the amount of tumors formed by RCC cells with CD73 shRNA was less than those with control shRNA, and the maximal tumor size was smaller correspondingly, indicating that tumor formation of cells with reduced CD73 expression was notably suppressed.

## DISCUSSION

In this study, we identified CD73 as a specific cell surface biomarker of ccRCC CSCs basing on our previous studies [26, 27] and discovered the association of CD73 intensive expression levels with ccRCC tumor formation by using siRNA technology. To the best of our knowledge, this represents the first identification of CD73 as a cell surface biomarker for ccRCC CSCs and also the first demonstration of the involvement of CD73 in ccRCC progression.

Previously, CD73 has been reported to be over-expressed in several types of cancer, and some studies have shown that tumor-derived CD73 is closely related to tumor development both *in vivo* and *in vitro* [32–34]. Differential expression of CD73 in RCC stem cells and non-stem cells has been noted [21, 35], but it has not been fully validated as a CSC marker for ccRCC. More recently, Katsuta *et al*. [36] identified CSCs basing on ALDH activity in pancreatic neuroendocrine tumor (pNET) clinical specimens and cell lines, and found that CD73 was overexpressed in ALDH^high^ cells, and inhibition of CD73 significantly attenuated *in vitro* sphere formation and cell motility, as well as *in vivo* tumor growth for ALDH^high^ cells. CD73 expression was also significantly correlated with invasive character observed in clinical tissue samples, and thus CD73 was proposed as a biomarker and potential therapeutic target for pNET CSCs. In our study, CD73 expression appears not to be associated with the expression of several common well-known CSC markers [37] (Supplementary Figure 4) in RCC. CD105, CD133 and CD24 only marginally coexpressed either in CD73^high^ or CD73^low^ cell population, but CD44 represented a major subpopulation in both two population cells. CD105/endoglin is not essential for tumor initiation in this study, but marks a rare, highly tumorigenic subpopulation of RCC tumor-initiating cells in some cell lines [38, 39]. The use of CD105 as a renal CSC marker was questioned in many studies showing that other putative subpopulations of cells with CSC-like properties were CD105^−^ [40, 41]. CD133 has been proposed to be a stem/progenitor marker in normal human kidney and RCC [21, 42–47]. However, the specificity of CD133 as a marker for stem/progenitor cells is a matter of debate, as it has been found on various differentiated adult epithelial cells, including renal tubular cells [15–48]. Galleggiante *et al*. [49] described and characterized a population of resident CD133+/CD24+/CTR2+ cancer stem cells in patients with ccRCC, nevertheless CD133+/CD24+ cells exist both in tubular adult renal progenitor cell and RCC derived cells. In addition, Varna *et al*. [50] identified a larger number of CD133/CXCR4-coexpressing cells in perinecrotic versus perivascular areas in RCC tissue. Moreover, Canis *et al*. [51] showed that stable transfection of CD133 in the human embryonic kidney 293 (HEK293) cell line induced tumor-initiating properties in these cells, and that HEK293 CD133^high^ transfectants, when injected into SCID mice, generated tumors with at least a 1000-fold increased frequency as compared with CD133^low^ cells. CD44 is well known to be a potential cancer stem cell marker in various cancers [52], *e.g*. in carcinomas of breast and ovary [53, 54]. Some researches [55, 56] demonstrated that both hypoxic and sunitinib-treatments resulted in up-regulation of CD44 expression in high-grade ccRCCs, and CD44 expression was stimulate by its up-stream molecule TNF-α. CD44 expression in ccRCC cells was not associated with clinical prognosis [21, 56], while the role of CD44 in chemotherapy resistance of ccRCC was confirmed [55]. Our data suggest that CD73 is a unique cell surface biomarker of ccRCC CSCs.

CD73 expression is induced by hypoxia [57] and hypoxia inducible factor-1 (HIF-1) [58], which implies that the microenvironment of malignant tumor elevates the expression of CD73. On the other hand, the suppression of CD73 inactive epidermal growth factor receptor and beta-catenin/cyclin D1 signaling pathways in human colorectal cancer cells [34] and also reduces the formation of ccRCC, which implies that CD73 is involved in tumor progression and can also be used as a target for tumor therapy. Obviously, the association of CD73 with ccRCC CSC remains ambiguous. CD73 is a biomarker for human mesenchymal stem cells (MSCs) [59, 60], which can also be isolated from the kidney and renal cancer cells [61, 62]. The cells originated in ccRCC are not definitively known whilst ccRCCs is commonly thought to arise from renal proximal epithelial cells [63], tubular epithelium is normally mitotically quiescent, but demonstrates a considerable regenerative capacity upon renal injury, the putative connection between renal stem cells and carcinomas is not clarified, since the understanding of the renal stem cell system is not complete yet [64]. The identification of CD73 as a biomarker of ccRCC CSC implies that the aggressiveness of ccRCC may be associated with an epithelial to mesenchymal transition (EMT). Evidence from various types of cancer suggest that the acquisition of EMT and induction of CSCs are interrelated [65, 66]. Kidneys are mesenchymal- originated and develop *via* biological including mesenchymal epithelial transition (MET). So, we propose that this process is reversed in the development of ccRCC, which results in EMT and dedifferentiation. However, this hypothesis requires further investigation in our future research.

The side population (SP) approach has been widely used to isolate CSCs from solid tumors [67–69] including RCC [13, 70]. There have been some discrepancies in the use of Rho-123 staining patterns to identify the subpopulation of CSCs [71, 72]. In contrast to other reports [71–73], our group has recently identified, from human RCC cell line 786-O a small subset of Rho^high^ cells have stem-like characteristics [26], and this result in according with our finding in clinical specimen. In several cancer cell lines where the Rho^low^ population is associated with CSC, we noticed that the majority of the cell population was Rho^low^, and the inhibition of P-glycoprotein (P-gp) with verapamil treatment did not influence the ratio between Rho^high^ and Rho^low^ (Supplementary Figure 5A). However, in 786-O RCC cell line, there was a smaller subpopulation of Rho^high^ cells, and verapamil treatment markedly increased the proportion of Rho^high^ subpopulation. Furthermore, we found that the Rho^high^ and CD73^high^ RCC cells displayed lower expression of P-gp than their counterpart (Supplementary Figure 5B). These findings indicate that ccRCC CSCs possess distinct characteristics from other CSCs even though the underlying mechanisms remain unknown.

In summary, we demonstrated the existence of CSCs in both cultured ccRCC cell line and tissues, and CD73 as a cell-surface biomarker for ccRCC CSC-like cells. This is mainly based on the finding that stem cell properties and tumor-initiating potential of these cells. CD73 is applicable for the prognosis of ccRCC and also can be served as a potential target for ccRCC treatment.

## MATERIALS AND METHODS

### Culture of RCC cells, tumor spheroids, and tumor subspheroids

A human ccRCC cell line, 786-O, was obtained from the Cell Bank of the Chinese Academy of Sciences (Shanghai, China). Monolayer adherent cells (MACs) were maintained as monolayer cultures in RPMI-1640 medium (Invitrogen, Eugene, OR) supplemented with 10% fetal bovine serum (FBS, Hyclone, Logan, UT), 100 IU/mL penicillin G and 100 μg/mL streptomycin and cultured at 37°C in a humidified 5% CO_2_ incubator. Tumor spheroids formed after changing the medium to serum-free RPMI-1640 medium (SFM) supplemented with mitogens, including 10 ng/mL basic fibroblast growth factor (bFGF), 20 ng/mL epidermal growth factor (EGF), 5 μg/mL insulin, and 0.4% bovine serum albumin (Sigma-Aldrich, St. Louis, MO). The cells were subsequently cultured in ultra-low-attachment 6-well plates (Corning, NY) at a density of 5,000 cells/well. The frequency of spheroid formation was determined by a limiting dilution assay, in which different numbers of cells (1–500) per well were plated into ultra-low-attachment 96-well plates. When primary tumor spheroids formed after 7–10 days, spheroid-forming cells (SFCs) were collected by gravity and enzymatically dissociated by incubation in Accutase (Invitrogen) for 5–10 min at room temperature, then subcultured at 1 × 10^3^ cells/mL in SFM supplemented with mitogens to test their capacity to regenerate spheroids.

### Rho-123 staining analysis

For rhodamine-123 (Rho-123) staining, cell suspensions at 10^6^ cells/mL were exposed to 0.1 μg/mL of Rho-123 (Sigma) for 20 min at 37°C in the dark. The cells were then washed and re-suspended in serum-enriched medium and kept at 37°C for another 30 min. After washing with phosphate buffered saline (PBS) containing 2% FBS, the cells were then stained with propidium iodide (2 μg/mL; Sigma) before flow-cytometric sorting to exclude dead cells. Control cells were incubated with 50 μM verapamil (Sigma) for 15 min at 37°C before Rho-123 dye addition to inhibit the ABCB1 transporter. Cell analysis and sorting were performed on a FACSCalibur flow cytometer (BD Bioscience, San Diego, CA).

### SILAC and MS quantitative analysis

Cells were cultured in SILAC^®^ RPMI-1640 medium, either containing 10% dialyzed fetal bovine serum and supplemented with 100 μg/mL ^13^C_6_^15^N_4_-L-arginine (Invitrogen), designated as ‘heavy’ medium, or containing 10% fetal bovine serum and supplemented with 100 μg/mL regular arginine (Invitrogen), designated as ‘light’ medium. Cultures were maintained for 2 weeks to achieve complete labeling of cellular proteins with ‘heavy’ or ‘light’ amino acids before sorting for Rho^high^ or Rho^low^ cells, respectively. Separate suspensions of sorted Rho^high^ and Rho^low^ cells were washed three times with ice-cold PBS and then suspended in 8 M urea containing a protease inhibitor cocktail (Roche, Basel, Switzerland) and sonicated. After centrifugation for 30 min at 20,000 × *g* in a bench-top centrifuge (Thermo Fisher Scientific, Waltham, MA), the supernatants were collected and kept at −80°C for analysis. Protein concentrations were measured using the Bradford method. Extracted protein samples from heavy Rho^high^ cells and light Rho^low^ cells were then combined at a 1:1 ratio. In-solution digestion was performed as follows. Briefly, 100 μg of protein mixture was dissolved in 8 M urea with 25 mM NH_4_HCO_3_ (Sigma), reduced with 10 mM DTT for 1 h, alkylated with 40 mM iodoacetamide in the dark for 45 min at room temperature, and then the iodoacetamide was quenched with 40 mM DTT for 30 min at room temperature. After diluting the 8 M urea with 25 mM NH_4_HCO_3_ to 1.6 M, sequence grade trypsin (Promega, Madison, WI) was added at a ratio of 1:30 and the protein mixture was digested at 37°C overnight. Tryptic digestion was stopped by the addition of formic acid to a 1% final concentration. Digests were centrifuged at 16,000 × *g* for 10 min prior to analysis. The supernatant was analyzed by 2D-LC on an LTQ Orbitrap XL (Thermo Fisher Scientific) [74]. The MS/MS data were searched against a human protein database downloaded from the NCBI database using the SEQUEST program (Thermo Fisher Scientific). The annotations of proteins were obtained from the Swiss-Prot and TrEMBL protein databases. For proteins without descriptions, annotations were obtained by searching the IPI, Swiss-Prot and TrEMBL protein database with BlastP for homologous proteins with descriptions. The PANTHER classification system was used for protein sorting with slight modification where a few protein groups with similar annotation were combined.

### Flow-cytometric analysis and cell sorting

Antibodies used were fluorescein isothiocyanate (FITC)-conjugated CD44, R-phycoerythrin (PE)-conjugated CD24, PE-conjugated CD105, PE-conjugated CD133 and PE-conjugated CD73. Cells were washed with a solution of PBS and 0.5% bovine serum albumin, centrifuged at 300 × *g*, and the supernatant was removed. The antibodies were added at a 1:10 dilution (5–50 μL) in PBS with 0.5% bovine serum albumin and incubated on ice in the dark for 30 min. Appropriate isotype controls were used for each antibody. The cells were then analyzed using a FACSCalibur flow cytometer with CellQuest software (BD Bioscience). At least 10,000 events were collected for each sample. Experiments were performed in triplicate with consistent results. Fluorescence-activated cell sorting was performed using standard protocols. Single cell suspensions were labeled with PE-conjugated antibody to CD73 (PharMingen, Becton Dickinson UK Ltd., Oxford, UK) for 30 min on ice. The cells were then analyzed as above.

### Proliferation assay and cell cycle analysis

Trypan blue staining (Invitrogen) was used to measure cell viability. Five hundred healthy 786-O SFCs and MACs or sorted cells were cultured with RPMI-1640 medium containing 10% FBS and the viability of the cells was measured using the 3-(4,5-dimethylthiazol-2-yl)-2,5- diphenyltetrazolium bromide (MTT) method (Sigma). The cell growth curves were drawn accordingly. For cell cycle analysis, SFCs and MACs were harvested and fixed with 70% ethanol for 24 h. Cells were washed with PBS, stained with 40 μg/mL propidium iodide and 1 μg/mL RNase (Sigma), and then analyzed on a FACSCalibur flow cytometer. The percentage of proliferating cells was calculated using the formula: (G2/M ^+^ S)/(G0/G1 ^+^ S ^+^ G2/M) × 100%.

### Chemo- and radio-sensitivity assays

The sensitivity of the 786-O parental and SFC cells to chemotherapeutic drugs was measured by MTT assay. Briefly, SFCs and MACs cells were seeded into 96-well plates (2000 cells/well) and 24 h later, various concentrations of mitomycin C (MMC) (Sigma) were added. Cell viability was evaluated after 2 days of drug treatment by MTT assay. Absorbance was measured at 490 nm. To determine the sensitivity of various samples to ionizing radiation, a routine colony-forming assay was used. Briefly, a small number of cells were cultured in RPMI-1640 with 10% FBS. After 6 h, the dishes were irradiated with X-rays (100 kVp, 5 mA, with no additional filtering; Faxitron-650, Faxitron Bioptics LLC, Tucson, AZ) at a dose rate of 1 Gy/min. After incubating for a further 10 days, the dishes were fixed and stained with 0.25% crystal violet, and colonies containing ≥ 50 cells were counted and used to calculate the surviving fraction. Data shown represent the mean of three independent experiments.

### *In vivo* tumorigenicity experiments

We used 6- to 8-week-old female nude mice and non-obese diabetic/severe combined immunodeficient (NOD/SCID) mice (Institute of Laboratory Animal Science, Chinese Academy of Medical Sciences, Beijing, China) for tumor xenograft assays. All mice were cared for in the Institute of Tumor, Gansu Academy of Medical Sciences, under the approval of the Institutional Review Board or the Animal Care and Use Committee. Before injection, cell viability was assessed with Trypan blue staining. To investigate tumorigenic capacity, 100 μL viable Rho^high^ and Rho^low^ cells suspended in serum-free medium were directly injected into the left and right inguen of the nude mouse. To compare tumorigenic capacity of monolayer adherent cells (MACs) and spheroids (SFCs), the 786-O parental and the fifth passage spheroid cells were each suspended in serum-free RPMI-1640 and mixed with 100 μL Matrigel (BD Biosciences, San Jose, CA) and subcutaneously injected into NOD/SCID mice at the inguinal site. To determine the tumor forming capacity of CD73^high^ and CD73^low^ cells, sorted cells suspended in serum-free medium were mixed with the same volume of Matrigel and injected into mice as above. To compare the tumor forming capacity of cells with or without CD73 knockdown, 5 × 10^4^, 5 × 10^3^, and 5 × 10^2^ of cells stably expressing CD73 shRNA or control shRNA cells were suspended in serum-free medium, mixed with the same volume of Matrigel and injected into nude mice. For comparison, cells expressing CD73 shRNA were injected into the left and those expressing control shRNA into right inguen of the same mouse. Tumor formation was monitored weekly after implantation. Tumor volume was measured with a caliper and calculated using the formula: v = L × l^2^ × 0.5 where “L” indicates the large diameter and “l” indicates the small diameter as previously described [75]. Animals were sacrificed after 12–16 weeks, and a proportion of the tumors were dissected and finely minced, washed four times with washing buffer (RPMI-1640 containing 200 IU/mL penicillin, 200 μg/mL streptomycin, 200 IU/mL nystatin, and 100 μg/mL gentamycin), digested with 5 mg/mL collagenase type II (Sigma) at 37°C for 2 h, and passed through a 40-μm filter to obtain a cell suspension for further experiments or inoculation into mice again. The remaining subcutaneous tumors were excised and fixed in 2% formaldehyde. Hematoxylin-eosin staining was performed on 5-μm paraffin-embedded sections following standard protocols [76].

### Western blotting analysis

Quantified protein lysates were separated on SDS-PAGE gels, transferred onto a polyvinylidene difluoride (PVDF) membrane (Millipore, Billerica, MA), and immunoblotted with the primary antibodies followed by incubation with a horseradish peroxidase-conjugated secondary antibody. β-actin or GADPH was used as a loading control. Antibodies used in this study were anti-β-actin (Santa Cruz Biotechnology, Santa Cruz, CA),, anti-GADPH (Santa Cruz Biotechnology), anti-P-glycoprotein (Abcam, Cambridge, MA), and anti-human CD73 (Abcam).

### Semiquantitative reverse transcription polymerase chain reaction (RT-PCR)

Cells were harvested in TRIzol reagent (Invitrogen) and total RNA was extracted following the manufacturer's protocol. RNA was quantitated by spectrophotometry at OD_260_. First strand cDNA was prepared using Oligo (dT)12–18 (Invitrogen) primers, 1.2 μg of total RNA, and SuperScript II RNase H Reverse Transcriptase (Invitrogen) according to the manufacturer's recommendations. The target cDNA was amplified using Platinum Taq DNA Polymerase (Invitrogen) for 28–30 cycles. Primer sequences are provided in Supplementary Table 1. Aliquots of 8 μL of the amplification products were separated by electrophoresis in 1.5% agarose gels, stained in 1 × TAE containing GelStar^®^ (Lonza, Basel, Switzerland), and then visualized under a UV transilluminator.

### Soft agar colony-forming assay

The soft agar colony formation assay was performed as previously described [26]. Briefly, triplicate 60 mm dishes were coated with a 1 mL base layer containing 0.70% agarose. On day 0, cells were dissociated and subcultured by layering cell suspensions at various cell densities in 0.5 mL culture medium containing 0.36% agarose onto the base coat. Culture dishes were refrigerated (4°C) for 15 min to set the agarose, brought to room temperature for 10 min, and then overlaid with 0.5 mL culture medium and incubated at 37°C. Colonies were counted under an inverted microscope at the indicated time points.

### Tissue samples

Primary human ccRCC tissues were obtained from surgically-removed tumor blocks from patients treated by either partial or radical nephrectomy for renal tumors at Lanzhou University Second Hospital between 2010 and 2012, with the approval of the Local Ethics Board and consent from patients. No patients had received radiotherapy or chemotherapy prior to surgery. Samples were obtained from the visible tumor as well as from grossly uninvolved, adjacent renal parenchyma. Cancers were examined and graded by pathologists at the Lanzhou University Second Hospital. Tumor specimens were finely minced with scissors and then digested by incubation for 1 h at 37°C in RPMI-1640 containing collagenase II (Sigma). After washing with medium containing 10% fetal calf serum (Life Technologies, Inc., Grand Island, NY), the cell suspension was forced through a graded series of meshes to separate the cell components from stroma and aggregates. Aliquots of the cell suspension were subjected to fluorescence-activated cell sorting to quantify the proportion of either Rho^high^ cells or CD73^high^ cells and subsequent experiments. Formalin-fixed, paraffin embedded samples of healthy renal tissue (*n* = 15) and ccRCC (*n* = 44) were obtained as described above.

### Immunohistochemistry

All tissues were preserved in 10% formalin, embedded in paraffin, then serially sectioned onto microscope slides with a thickness of 4 μm, deparaffinized in xylene, cleared in a graded ethanol series, and heat-treated for 20 min in Antigen Unmasking Solution in a food steamer. Sections were immunostained with monoclonal antibody clone 2B6 to human CD73 (1:400, Abcam) using avidin-biotin-complex-peroxidase. Negative control slides were prepared by omitting the primary antibody. The chromogen reaction was carried out with diaminobenzidine and slides were counterstained with hematoxylin. Staining was scored according to the method reported previously [77]. Two pathologists performed the evaluation of the staining in a blinded manner. The CD73 score was measured by applying the following formula: (mean percentage) × (intensity ^+^ 1, range 0–400). The intensity of immunostaining and the proportion of positive cells were roughly graded into four scores (0, 1, 2 and 3). Score 3 was defined as maximum intensity of immunostaining throughout the section while score 0 implied that staining was absent throughout the specimen.

### CD73 knockdown with shRNA

Cells were cultured at a density of 1 × 10^5^ cells per well in a 12-well plate for 48 h before infection. For cell infection, cells were incubated with 10 MOI of CD73 shRNA lentiviruses (Santa Crutz) and control cells were infected with 10 MOI of control shRNA (Santa Crutz, sc-108084) for 24 h according to the manufacturer's instruction. A transfection efficiency more than 90% was achieved basing on the evaluation with GFP lentiviruses (Santa Crutz, sc-108080) with the same infection protocol. Stable transfection clones were selected by using the medium containing 1 mM puromycin. The expression of CD73 protein was confirmed by Western blot assay.

### Statistical analysis

All values in the figures and text are shown as mean ± SEM. Statistical analyses were performed using the SPSS statistical software package 16.0 (SPSS/PC^+^, SPSS Inc., Chicago, IL). Any significant differences in mean values were evaluated by Student's *t*-test. A two-sided *P* < 0.05 was accepted as significant.

## SUPPLEMENTARY MATERIALS FIGURES AND TABLES



## References

[1] Rini BI, Campbell SC, Escudier B (2009). Renal cell carcinoma. Lancet.

[2] Koul H, Huh JS, Rove KO, Crompton L, Koul S, Meacham RB, Kim FJ (2011). Molecular aspects of renal cell carcinoma: a review. Am J Cancer Res.

[3] Siegel RL, Miller KD, Jemal A (2015). Cancer statistics, 2015. CA Cancer J Clin.

[4] Najjar YG, Rini BI (2012). Novel agents in renal carcinoma: a reality check. Ther Adv Med Oncol.

[5] Reya T, Morrison SJ, Clarke MF, Weissman IL (2001). Stem cells, cancer, and cancer stem cells. Nature.

[6] Driessens G, Beck B, Caauwe A, Simons BD, Blanpain C (2012). Defining the mode of tumour growth by clonal analysis. Nature.

[7] Chen J, Li Y, Yu TS, McKay RM, Burns DK, Kernie SG, Parada LF (2012). A restricted cell population propagates glioblastoma growth after chemotherapy. Nature.

[8] Schepers AG, Snippert HJ, Stange DE, van den Born M, van Es JH, van de Wetering M, Clevers H (2012). Lineage tracing reveals Lgr5+ stem cell activity in mouse intestinal adenomas. Science.

[9] Rosen JM, Jordan CT (2009). The increasing complexity of the cancer stem cell paradigm. Science.

[10] Khan MI, Czarnecka AM, Helbrecht I, Bartnik E, Lian F, Szczylik C (2015). Current approaches in identification and isolation of human renal cell carcinoma cancer stem cells. Stem Cell Research and Therapy.

[11] Jordan CT (2004). Cancer stem cell biology: from leukemia to solid tumors. Curr Opin Cell Biol.

[12] Lichner Z, Saleh C, Subramaniam V, Seivwright A, Prud’homme GJ, Yousef GM (2014). miR-17 inhibition enhances the formation of kidney cancer spheres with stem cell/tumor initiating cell properties. Oncotarget.

[13] Ueda K, Ogasawara S, Akiba J, Nakayama M, Todoroki K, Sanada S, Suekane S, Noguchi M, Matsuoka K, Yano H (2013). Aldehyde dehydrogenase 1 identifies cells with cancer stem cell-like properties in a human renal cell carcinoma cell line. PloS One.

[14] Yamashita M, Hirohashi Y, Torigoe T, Kusumoto H, Murai A, Imagawa T, Sato N (2016). Dnajb8, a member of the heat shock protein 40 family has a role in the tumor initiation and resistance to docetaxel but is dispensable for stress response. PLoS One.

[15] Kim K, Ihm H, Ro JY, Cho YM (2011). High-level expression of stem cell marker CD133 in clear cell renal cell carcinoma with favorable prognosis. Oncol Lett.

[16] Costa WH, Rocha RM, Cunha IW, Fonseca FP, Guimaraes GC, Zequi Sde C (2012). CD133 immunohistochemical expression predicts progression and cancer-related death in renal cell carcinoma. World J Urol.

[17] D’Alterio C, Cindolo L, Portella L, Polimeno M, Consales C, Riccio A, Cioffi M, Franco R, Chiodini P, Carteni G, Mirone V, Longo N, Marra L (2010). Differential role of CD133 and CXCR4 in renal cell carcinoma. Cell Cycle.

[18] Hasmim M, Bruno S, Azzi S, Gallerne C, Michel JG, Chiabotto G, Lecoz V, Romei C, Spaggiari GM, Pezzolo A, Pistoia V, Angevin E, Gad S (2016). Isolation and characterization of renal cancer stem cells from patient-derived xenografts. Oncotarget.

[19] Wu Y, Wu PY (2009). CD133 as a marker for cancer stem cells: progresses and concerns. Stem Cells Dev.

[20] Matak D, Szymanski L, Szczylik C, Sledziewski R, Lian F, Bartnik E, Sobocinska A, Czarnecka AM (2015). Biology of renal tumour cancer stem cells applied in medicine. Contemporary Oncology.

[21] Bussolati B, Dekel B, Azzarone B, Camussi G (2013). Human renal cancer stem cells. Cancer Letters.

[22] Gedye C, Sirskyj D, Lobo NC, Evans A, Fleshner N, Robinette M, Zlotta A, Hamilton R, Kulkarni G, Finelli A, Gallie BL, Jewett MAS, Ailles L (2013). VHL-mutant renal cell carcinomas contain cancer cells with mesenchymal phenotypes. Journal of Clinical Oncology.

[23] Zhong Y, Guan K, Guo S, Zhou C, Wang D, Ma W, Zhang Y, Li C, Zhang S (2010). Sphere derived from the human SK-RC-42 renal cell carcinoma cell line are enriched in cancer stem cells. Cancer Lett.

[24] Gassenmaier M, Chen D, Buchner A, Henkel L, Schiemann M, Mack B, Schendel DJ, Zimmermann W, Pohla H (2013). CXC chemokine recepter 4 is essential for maintenance of renal cell carcinoma-initiating cells and predicts metastasis. Stem Cells.

[25] Saroufim A, Messai Y, Hasmim M, Rioux N, Iacovelli R, Verhoest G, Bensalah K, Patard JJ, Albiges L, Azzaroune B, Escudier B, Chouaib S (2014). Tumoral CD105 is a novel independent prognostic marker for prognosis in clear cell renal cell carcinoma. British Journal of Cancer.

[26] Lu JZ, Cui Y, Zhu JY, He JP, Zhou GM, Yue ZJ (2013). Biological characteristics of Rh123high stem-like cells in a side population of 786-O renal carcinoma cells. Oncol Lett.

[27] Yu Y, Wang W, Song L, Hu WT, Dong C, Pei HL, Zhou GM, Yue ZJ (2015). Ecto-5′-nucleotidase expression is associated with the progression of renal carcinoma. Oncol Lett.

[28] Lundholm L, Haag P, Zong D, Juntti T, Mork B, Lewensohn R, Viktorsson K (2013). Resistance to DNA-damaging treatment in non-small cell lung cancer tumor-initiating cells involves reduced DNA-PK/ATM activation and diminished cell cycle arrest. Cell Death Dis.

[29] Abdullah LN, Chow EK (2013). Mechanisms of chemoresistance in cancer stem cells. Clin Transl Med.

[30] Chang CH, Zhang M, Rajapakshe K, Coarfa C, Edwards D, Huang S, Rosen JM (2015). Mammary stem cells and tumor-initiating cells are more resistant to apoptosis and exhibit increased DNA repair activity in response to DNA damage. Stem Cell Reports.

[31] Cojoc M, Mabert K, Muders MH, Dubrovska A (2015). A role for cancer stem cells in therapy resistance: cellular and molecular mechanisms. Semin Cancer Biol.

[32] Wu XR, He XS, Chen YF, Yuan RX, Zeng Y, Lian L, Zou YF, Lan N, Wu XJ, Lan P (2012). High expression of CD73 as a poor prognostic biomarker in human colorectal cancer. J Surg Oncol.

[33] Supernat A, Markiewicz A, Welnicka-Jaskiewicz M, Seroczynska B, Skokowski J, Sejda A, Szade J, Czapiewski P, Biernat W, Zaczek A (2012). CD73 expression as a potential marker of good prognosis in breast carcinoma. Appl Immunohistochem Mol Morphol.

[34] Wu R, Chen Y, Li F, Li W, Zhou H, Yang Y, Pei Z (2016). Effect of CD73 on human colorectal cancer cell growth *in vivo* and *in vitro*. Oncol Rep.

[35] Bruno S, Bussolati B, Grange C, Collino F, Graziano ME, Ferrando U, Camussi G (2006). CD133 Renal Progenitor Cells Contribute to Tumor Angiogenesis. Am J Pathol.

[36] Katsuta E, Tanaka S, Mogushi K, Shimada S, Akiyama Y, Aihara A, Matsumura S, Mitsunori Y, Ban D, Ochiai T, Kudo A, Fukamachi H, Tanaka H (2016). CD73 as a therapeutic target for pancreatic neuroendocrine tumor stem cells. Int J Oncol.

[37] Peired AJ, Sisti A, Romagnani P (2016). Renal Cancer Stem Cells: Characterization and Targeted Therapies. Stem Cells Int.

[38] Barbara NP, Wrana JL, Letarte M (1999). Endoglin is an accessory protein that interacts with the signaling receptor complex of multiple members of the transforming growth factor-beta superfamily. J Biol Chem.

[39] Sanz-Rodriguez F, Guerrero-Esteo M, Botella LM, Banville D, Vary CP, Bernabéu C (2004). Endoglin regulates cytoskeletal organization through binding to ZRP-1, a member of the Lim family of proteins. J Biol Chem.

[40] Ueda K, Ogasawara S, Akiba J, Nakayama M, Todoroki K, Ueda K, Sannada S, Suekane S, Noguchi M, Matsuoka K, Yano H (2013). Aldehyde dehydrogenase 1 identifies cells with cancer stem cell-like properties in a human renal cell carcinoma cell line. PLoS One.

[41] Gassenmaier M, Chen D, Buchner A, Henkel L, Schiemann M, Mack B, Schendel DJ, Zimmermann W, Pohla H (2013). CXC chenokine receptor 4 is essential for maintenance of renal cell carcinoma-initiating cells and predicts metastasis. Stem Cells.

[42] Bussolati B, Brossa A, Camussi G (2011). Resident stem cells and renal carcinoma. Int J Nephrol.

[43] Bussolati B, Bruno S, Grange C, Buttiglieri S, Deregibus MC, Cantino D, Camussi G (2005). Isolation of renal progrnitor cells from adult human kidney. Am J Pathol.

[44] Neuzil J, Stantic M, Zobalova R, Chladova J, Wang X, Prochazka L, Dong L, Andera L, Ralph SJ (2007). Tumour-initiating cells vs. cancer ‘stem’ cells and CD133: what’s in the name?. Biochem Biophys Res Commun.

[45] Varna M, Gapihan G, Feugeas JP, Ratajczak P, Tan S, Ferreira I, Leboeuf C, Setterblad N, Duval A, Verine J, Germain S, Mongiat-Artus P, Janin A (2015). Stem cells increase in numbers in perinecrotic areas in human renal cancer. Clin Cancer Res.

[46] Ward HH, Romero E, Welford A, Pickett G, Bacallao R, Gattone VH, SA Ness, Wandinger-Ness A, Roitbak T (2011). Adult human CD133/1(+) kidney cells isolated from papilla integrate into developing kidney tublules. Biochim Biophys Acta.

[47] Zhang Y, Sun B, Zhao X, Liu Z, Wang X, Yao X, Dong X, Chi J (2013). Clinical significances and prognostic value of cancer stem-like cells markers and vasculogenic mimicry in renal cell carcinoma. J Surg Oncol.

[48] Florek M, Haase M, Marzesco AM, Freund D, Ehninger G, Huttner WB, Corbeil D (2005). Prominin-1/CD133, a neural and hematopoietic stem cell marker, is expressed in adult human differentiated cells and certain types of kidney cancer. Cell Tissue Res.

[49] Galleggiante V, Rutigliano M, Sallustio F, Ribatti D, Ditonno P, Bettocchi C, Selvaggi FP, Lucarelli G, Battaglia M (2014). CTR2 identifies a population of cancer cells with stem cell-like features in patients with clear cell renal cell carcinoma. J Urol.

[50] Varna M, Gapihan G, Feugeas JP, Ratajczak P, Tan S, Ferreira I, Leboeuf C, Setterblad N, Duval A, Verine J, Germain S, Mongiat-Artus P, Janin A (2015). Stem cells increase in numbers in perinecrotic areas in human renal cancer. Clin Cancer Res.

[51] Canis M, Lechner A, Mack B, Zengel P, Laubender RP, Koehler U, Heissmeyer V, Gires O (2013). CD133 induces tumour-initiating properties in HEK293 cells. Tumour Biol.

[52] Zoller M CD44: can a cancer-initiating cell profit from an abundantly expressed molecule?. Nat Rev Cancer.

[53] Bhat-Nakshatri P, Appaiah H, Ballas C, Pick-Franke P, Goulet R, Badve S, Srour EF, Nakshatri H (2010). SLUG/SNAI2 and tumor necrosis factor generate breast cells with CD44+/CD24- phnotype. BMC Cancer.

[54] Muthukumaran N, Miletti-Gonzalez KE, Ravindranath AK, Rodriguez-Rodriguez L (2006). Tumor necrosis factor-alpha differentially modulates CD44 expression in ovarian cancer cells. Mol Cancer Res.

[55] Mikami S, Mizuno R, Kosaka T, Saya H, Oya M, Okada Y (2015). Expression of TNF-alpha and CD44 is implicated in poor prognosis, cancer cell invasion, metastasis and resistance to the sunitinib treatment in clear cell renal cell carcinomas. Int J Cancer.

[56] Ma C, Komohara Y, Ohnishi K, Shimoji T, Kuwahara N, Sakumura Y, Matsuishi K, Fujiwara Y, Motoshima T, Takahashi W, Yamada S, Kitada S, Fujimoto N (2016). Infiltration of tumor-associated macrophages is involved in CD44 expression in clear cell renal cell carcinoma. Cancer Sci.

[57] Colgan SP, Eltzschig HK, Echle T, Thompson LF (2006). Physiological roles for ecto-5′-nucleotidase (CD73). Purinergic Signaling.

[58] Synnestvedt K, Furuta GT, Comerford KM, Louis N, Karhausen J, Eltzschig HK, Hansen KR, Thompson LF, Colgan SP (2002). Ecto-5′-nucleotidase (CD73) regulation by hypoxia-inducible factor-1 (HIF-1) mediates permeability changes in intestinal epithelia. J Clin Invest.

[59] Odaba S, Sayar F, Guven G, Yanikkaya-Demirel G, Piskin E (2008). Separation of mesenchymal stem cells with magnetic nanosorbents carrying CD105 and CD73 antibodies in flow-through and batch systems. J Chromat B.

[60] Mahmood A, Harkness L, Abdallah BM, Elsafadi M, Al-Nbaheen MS, Aldahmash A, Kassem M (2012). Derivation of stromal (skeletal and mesenchymal) stem-like cells from human embroyonic stem cells. Stem Cells Dev.

[61] Semedo P, Correa-Costa M, Antonio Cenedeze M, Maria Avancini Costa Malheiros D, Antonia dos Reis M, Shimizu MH, Seguro AC, Pacheco-Silva A, Saraiva Camara NO (2009). Mesenchymal stem cells attenuate renal fibrosis through immune modulation and remodeling properties in a rat remnant kidney model. Stem Cells.

[62] Royer-Pokora B, Busch M, Beier M, Duhme C, de Torres C, Mora J, Brandt A, Royer HD (2010). Wilms tumor cells with WT1 mutations have characteristic features of mesenchymal stem cells and express molecular markers of paraxial mesoderm. Human Mol Genet.

[63] Cohen HT, McGovern FJ (2005). Renal-cell carcinoma. N Engl J Med.

[64] Axelson H, Johansson ME (2013). Renal stem cells and their implications for kidney cancer. Semin Cancer Biol.

[65] Scheel C, Weinberg R (2012). Cancer stem cells and epithelial-mesenchymal transition: concepts and molecular links. Semin Cancer Biol.

[66] Marie-Egyptienne DT, Lohse I, Hill RP (2013). Cancer stem cells, the epithelial to mesenchymal transition (EMT) and radioresistance: potential role of hypoxia. Cancer Letters.

[67] Hirschmann-Jax C, Foster AE, Wulf GG, Nuchtern JG, Jax TW, Gobel U, Goodell MA, Brenner MK (2004). A distinct „side population“ of cells with high drug efflux capacity in human tumor cells. Proc Natl Acad Sci USA.

[68] Golebiewska A, Brons NH, Bjerkvig R, Niclou SP (2011). Critical appraisal of the side population assay in stem cell and cancer stem cell research. Cell Stem Cell.

[69] Broadley KW, Hunn MK, Farrand KJ, Price KM, Grasso C, Miller RJ, Hermans IF, McConnell MJ (2011). Side population is not necessary or sufficient for a cancer stem cell phenotype in glioblastoma multiforme. Stem Cells.

[70] Huang B, Huang YJ, Yao ZJ, Chen X, Guo SJ, Mao XP, Wang DH, Chen JX, Qiu SP (2013). Cancer stem cell-like side population cells in clear cell renal cell carcinoma cell line 769P. PLoS One.

[71] Liu WH, Qian NS, Li R, Dou KF (2010). Replacing Hoechst33342 with rhodamine123 in isolation of cancer stem-like cells from the MHCC97 cell line. Toxicol *In Vitro*.

[72] Donnenberg VS, Meyer EM, Donnenberg AD (2009). Measurement of multiple drug resistance transporter activity in putative cancer stem/progenitor cells. Methods Mol Biol.

[73] Wang Y, Hao D, Stein WD, Yang L (2006). A kinetic study of Rhodamine123 pumping by P-glycoprotein. Biochimica et biophysica acta.

[74] Delahunty C, Yates JR (2005). Protein identification using 2D-LC-MS/MS. Methods.

[75] Wang ML, Pan CM, Chiou SH, Chen WH, Chang HY, Lee OK, Hsu HS, Wu CW (2012). Oncostatin m modulates the mesenchymal-epithelial transition of lung adenocarcinoma cells by a mesenchymal stem cell-mediated paracrine effect. Cancer Res.

[76] Lenartowicz M, Windak R, Tylko G, Kowal M, Styrna J (2010). Effects of copper supplementation on the structure and content of elements in kidneys of mosaic mutant mice. Biol Trace Elem Res.

[77] Mikami S, Oya M, Mizuno R, Murai M, Mukai M, Okada Y (2006). Expression of Ets-1 in human clear cell renal cell carcinomas: implications for angiogenesis. Cancer Sci.

