# Supplementary material for: Ecto-5′-nucleotidase (CD73) is a biomarker for clear cell renal carcinoma stem-like cells
Source: Oncotarget. 2017 Mar 29;8(19):31977–92. doi: 10.18632/oncotarget.16667 (PMC5458263; doi:10.18632/oncotarget.16667)
Supplement: Supplementary file 1 [file oncotarget-08-31977-s001.pdf]

Ecto-5′-nucleotidase (CD73) is a biomarker for clear cell renal carcinoma stem-like cells

Supplementary Materials

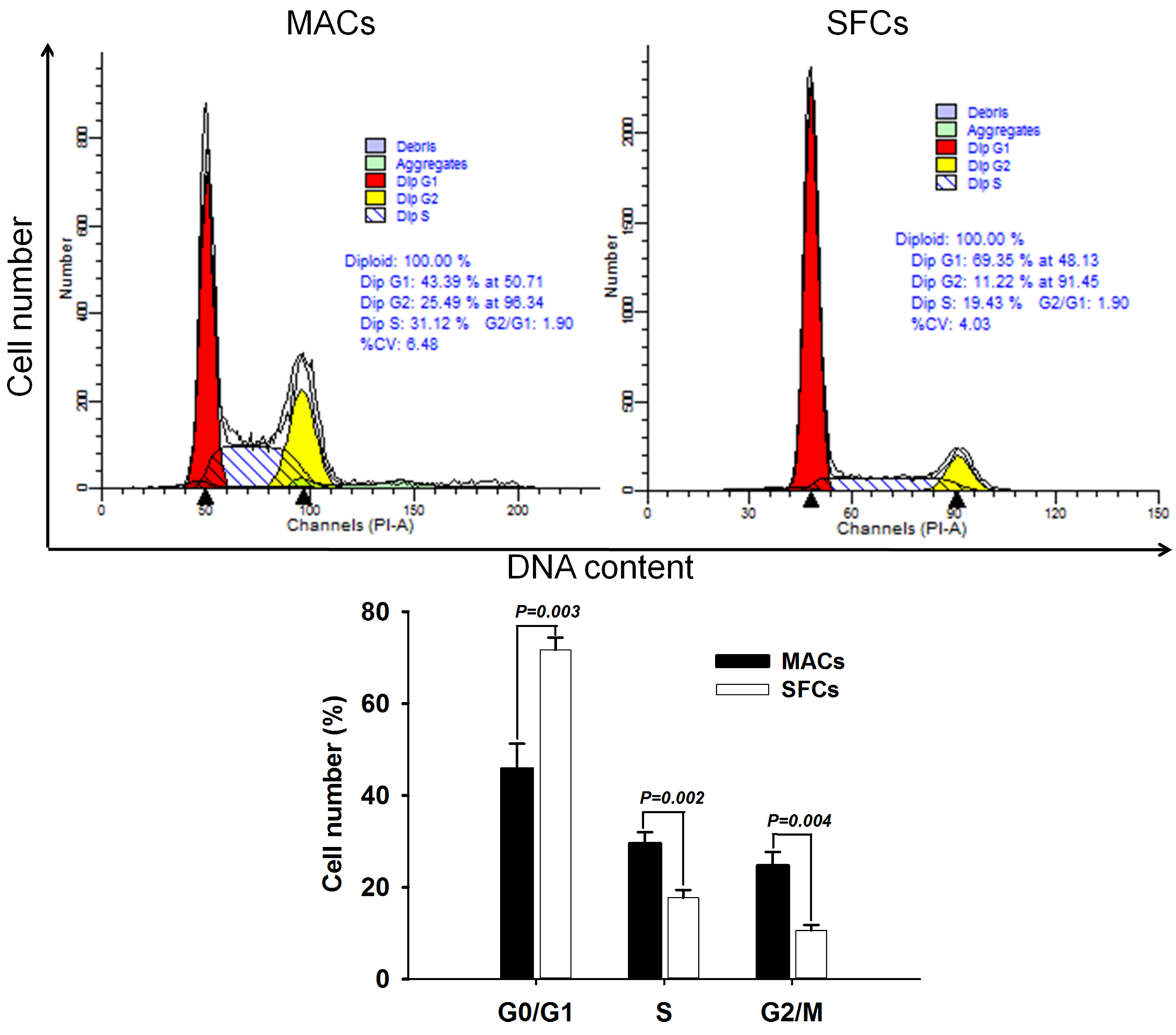

Supplementary Figure 1: Cell cycle of MACs and SFCs. Cells were stained with DNA staining solution, and analyzed by fluorescence-activated cell sorting. The percentage of cells in the G_0/G_1, S and G_2/M phases of the cell cycle was expressed as the mean ± standard errors of triplicate samples. *P* values was given.

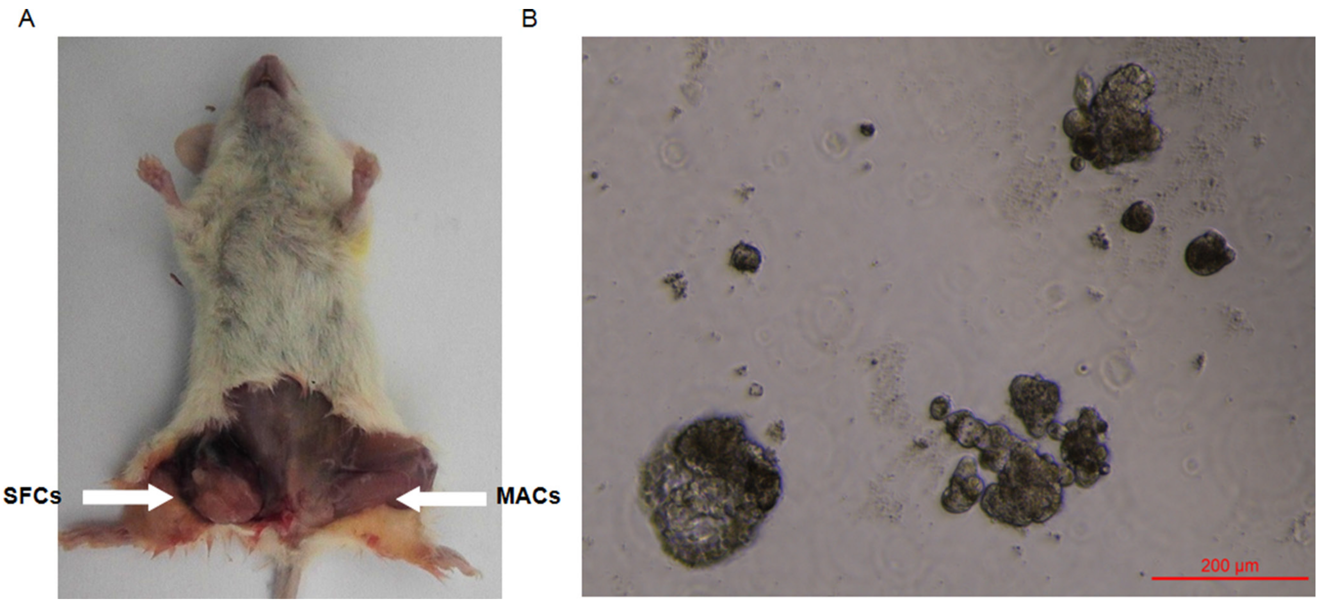

Supplementary Figure 2: Cells derived from xenograft tumor formed spheroids again in serum-free medium. (**A**) Representative formation of xenograft tumors by subcutaneous injection of 500 monolayer 786-O cells (labeled as MACs on the right flank) and by 500 resuspended spheroid cells (labeled as SFCs on the left flank) into inguens of the same NOD/SCID mouse. (**B**) When mice were sacrificed 120 days after cell inoculation, tumor were digested to single cell suspension and cultured in serum-free medium.

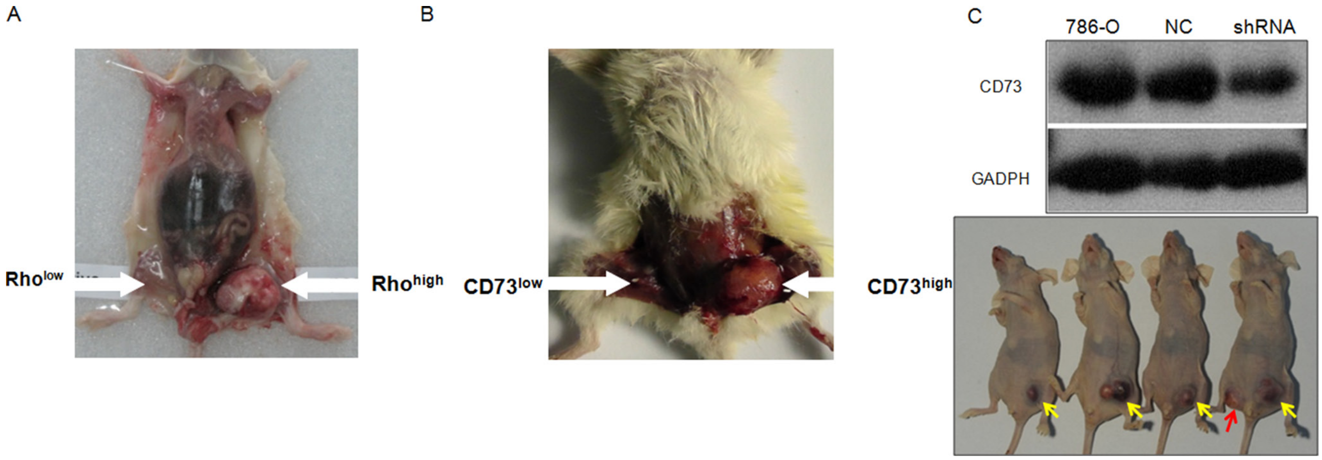

Supplementary Figure 3: Representative tumor formation in Xenograft assay. (**A**) Representative tumor formation in NOD/SCID mice injected with 10^6 Rho^{high} and Rho^{low} cells. (**B**) A representative xenograft tumor formed in NOD/SCID mice after injection with 500 CD73low cells and CD73high cells into the left and right inguens of the same NOD/SCID mouse. This picture was taken 4 months after injection. (**C**) Western blot of CD73 expression suppressed with or without shRNA. NC, control shRNA; shRNA, shRNA for CD73. GAPDH was used as a loading control. Representative xenograft tumors formed in athymic nude mice after injection with 50,000 cells transfected with CD73 shRNA or control shRNA into the left and right inguens, respectively into the same mouse. The picture was taken 110 days after injection. Yellow arrows indicate tumors formed by cells transfected with control RNA and red arrow indicates the tumor formed by cells transfected with CD73 shRNA.

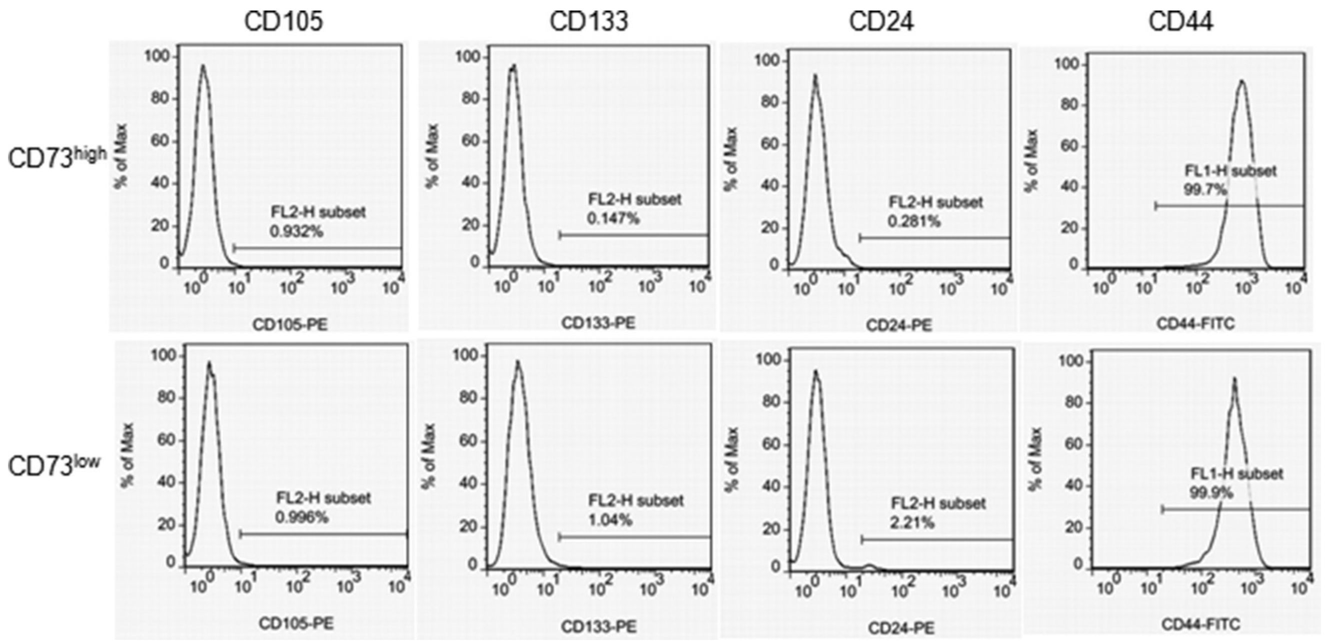

Supplementary Figure 4: Expression of previously identified CSC biomarkers in CD73^{high} and CD73^{low} cells. Flow cytometric profiles of a panel of CSC markers are shown. One representative result of three independent experiments is shown.

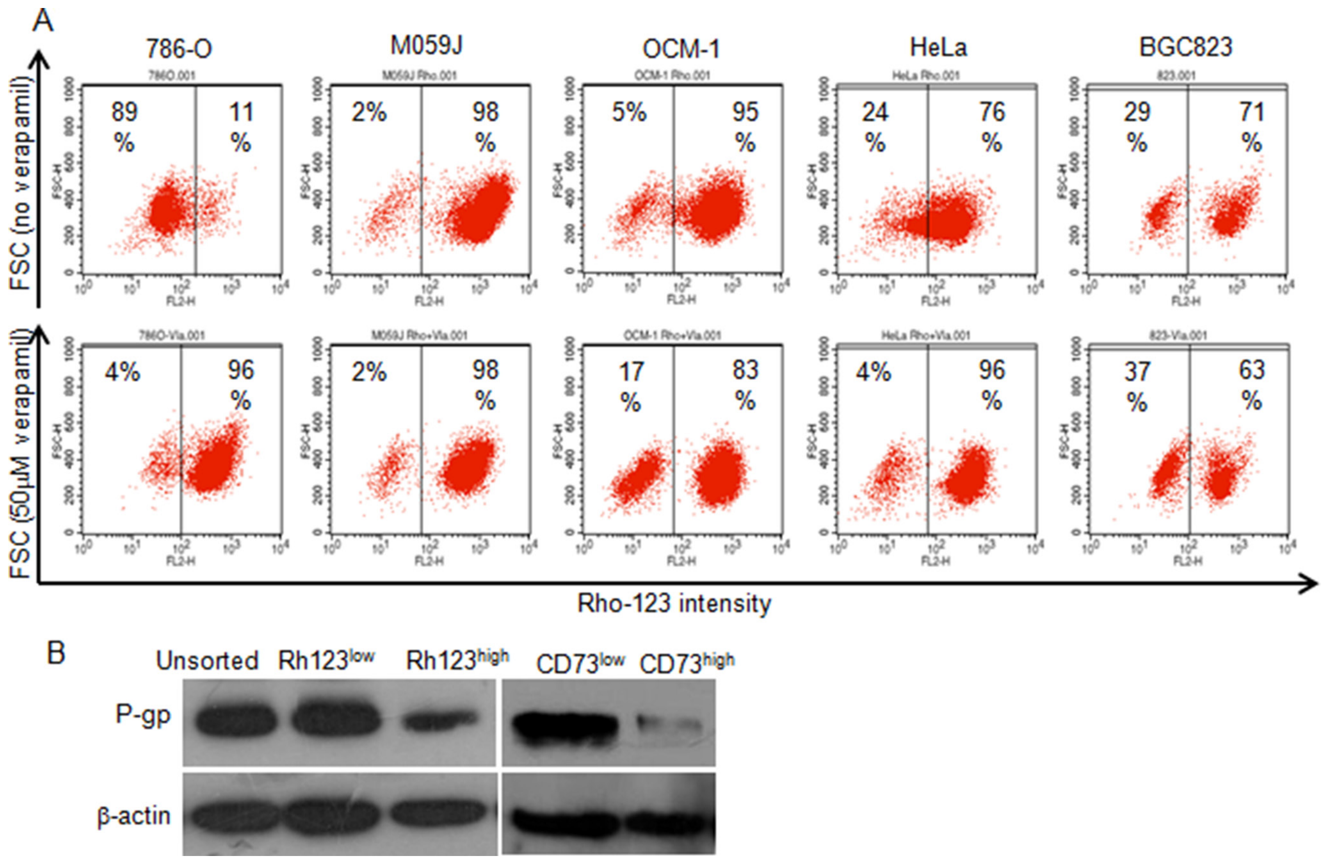

Supplementary Figure 5: Rh123 staining pattern in CCRCC differs from other cancer cell lines. (**A**) Flow cytometry profiles of human CCRCC cell line 786-O, glioma cell line M059J, melanoma cell line OCM-1, cervical carcinoma cell line HeLa, and gastric carcinoma cell line BGC823. All cells were incubated with Rh123 in the absence and presence of Verapamil (50 mM), which blocks Rh123 exclusion from cells. (**B**) Detection of P-gp in subpopulation of 786-O cells by Western blotting. β-actin was used as a loading control.

Supplementary Table 1: Primers sequences for RT-PCR

Genes	Primer sequences
β-actin	5′-AAGAGAGGCATCCTCACCCT-3′<br>5′-TACATGGCTGGGGTGTTGAA-3′
GAPDH	5′-CATGGCCTTCCGTGTTCCTA-3′<br>5′-GCGGCACGTCAGATCCA-3′
β-catenin	5′-ACTGGCAGCAACAGTCTTACC-3′<br>5′-TTTGAAGGCAGTCTGTCGTAAT-3′
BMI	5′-GGAGACCAGCAAGTATTGTCCTTTTG-3′<br>5′-CATTGCGCTGGGCATCGTAAG-3′
Oct3/4	5′-CGACCATCTGCCGCTTTGAG-3′<br>5′-CCCCCTGTCCCCCATTCCTA-3′
NANOG	5′-CAACTGGCCGAAGAATAGCA-3′<br>5′-GCAGGAGAATTTGGCTGGAA-3′
